# Comparing Perceptions of ChatGPT Use in Health Attitude Contexts Among Users and Nonusers: Cross-Sectional Study

**DOI:** 10.2196/79276

**Published:** 2026-04-27

**Authors:** Hessah Al Suwaidan, Arwa Althumairi, Saja A Al-Rayes, Khalid Alkhurayji

**Affiliations:** 1 Health Information Management and Technology Department, College of Public Health, Imam Abdulrahman Bin Faisal University, Dammam, Saudi Arabia; 2 Research, Statistics, and Information Department, Saudi Central Board for Accreditation of Healthcare Institutions, Wadi Alsaheya, Riyadh, 12264, Saudi Arabia, 966596181916

**Keywords:** artificial intelligence, AI, ChatGPT, health attitude, health informatics, Saudi Arabia

## Abstract

**Background:**

In light of the growing use of artificial intelligence (AI) in health care, individuals’ access to and use of health information are transforming. ChatGPT, an AI chatbot, provides immediate responses to health queries, with the potential to influence health-related attitudes, thereby raising concerns related to privacy, reliability, and security.

**Objective:**

This study aimed to investigate the perceived usefulness, risks, anxiety, and social influence of ChatGPT on health attitudes among users and nonusers in Saudi Arabia.

**Methods:**

A cross-sectional study was conducted using an online survey based on a validated tool. In total, 337 participants aged 18 years and older responded to questions assessing their perceptions of ChatGPT on health-related attitudes.

**Results:**

Data showed that 76.1% (194/255) of the respondents used ChatGPT, with the majority being younger and more highly educated. The main uses for health-related purposes were health education (43/194, 22.2%) and physical activity guidance (31/194, 16%). The analysis showed that users considered ChatGPT useful for health-related decisions, with 45.9% (89/194) finding it easy to learn and use, but concerns about privacy (106/194, 54.7%) and reliability (87/194, 44.9%) remained. Among nonusers, security risks (39/61, 63.9%) were the major barrier to using AI-based tools for health purposes, and 68.9% (42/61) found such tools attractive and engaging. There were no statistically significant differences between users and nonusers across all examined sociodemographic characteristics (*P*>.05).

**Conclusions:**

The study established the potential of ChatGPT in improving health decision-making and revealed cultural, privacy, and trust issues that may affect its implementation. These findings underscore the importance of strengthening the security of AI-based applications to enhance public acceptability of related health policies and to support the safe integration of tools such as ChatGPT into the health care system.

## Introduction

Over recent years, artificial intelligence (AI) has revealed significant potential in health care, particularly in education, research, decision-making, and teleconsultation [1,2]. The paradigm shift associated with this technology suggests that AI can address highly challenging problems and draw from enormous volumes of health-related information [3]. There have been affirmative advancements in interventions, public health screening, and program design, enhancing its use and promising to lead to more efficient health care systems [4,5].

One advanced AI tool is ChatGPT, a conversational agent developed by OpenAI, designed using natural language processing to deliver human-like conversational responses [6]. After its launch in late 2022, ChatGPT has been widely used, with 1 million users in the first week and 100 million in January 2023 [7]. The use of ChatGPT in health care practice has revealed approximately 70% clinical decision-making accuracy [8,9]. ChatGPT supports symptom screening, health optimization, resource use, and reduction of the load on the health care workforce [6,10].

Previous investigations have demonstrated that AI-generated tools can shape health behaviors and health care services [1]. According to a study conducted in the United Arab Emirates, positive attitudes toward ChatGPT were linked with lower perceived risks, lower anxiety, and higher scores in attitudes toward technology and social influence. Furthermore, a positive attitude was associated with Arab nationality and male sex [11]. Nonetheless, in Taiwan, use of ChatGPT was lower among individuals without prior experience with an AI tool but higher among male participants and those with higher educational attainment [12]. However, in Jordan, the investigation highlighted the need to consider perceptions of risk, usefulness, ease of use, and attitudes toward technology [13].

More than 23 million people across Europe use technology to make health-related decisions, and most use health applications [14]. In Saudi Arabia, AI implementation in health care, used through telemedicine and other online health applications, reveals a new trend toward the use of technology within the Saudi health system [15,16].

Although the adaptation of clinical procedures and productivity may benefit from the use of ChatGPT [6,17], additional research is required to investigate the health attitudes across Saudi Arabia to capture the cultural, linguistic, and privacy-related concerns [18,19].

The aim of this study was to compare perceptions of ChatGPT use by examining its perceived usefulness, associated risks, anxiety levels, and social influence on health attitudes among users and nonusers.

## Methods

### Research Design and Setting

This analytical cross-sectional study was conducted in Saudi Arabia to compare perceptions of ChatGPT, including perceived usefulness, perceived risk, anxiety, and social influence, on health attitudes among ChatGPT users and nonusers.

### Study Population and Sample Size Calculation

In total, 337 participants aged 18 years or older, residing in Saudi Arabia and having internet access, were recruited through an online survey. The single-population proportion formula was used to determine the required sample size, with a 95% CI (Z) and a 5% margin of error (E). After reviewing the existing literature to determine the smallest sample size (p), we found a study that reported a positive health care–related view of ChatGPT prevalence of approximately 70% [20]. Using the following formula, the minimum required sample size was calculated to be 323:


$$n=\frac{\left(Z^{2}\right)\times p\times \left(1-p\right)}{E^{2}}=\;\frac{{\left(1.96\right)}^{2}\times 0.70\times \left(1-0.70\right)}{\left(0.05\right){\;}^{2}}\approx 323.$$

The sample size was chosen to estimate the overall prevalence and characteristics of ChatGPT use within the target population, rather than to aim for an equal number of users and nonusers. In this study, the participants were classified into two groups: (1) ChatGPT users, defined as respondents who reported having used ChatGPT, and (2) ChatGPT nonusers, defined as respondents who had not used ChatGPT but were aware of and familiar with it (eg, through media exposure, discussions in academic classes, or peers). This approach ensured that all participants included in the analysis had sufficient awareness of ChatGPT to provide meaningful responses.

The inclusion criteria were participants aged 18 years or older, living in Saudi Arabia, and having access to the internet. Participants who reported no awareness or familiarity with ChatGPT were excluded from the analysis. This classification resulted in an analytic sample of 255 participants (n=194, 76.1% users and n=61, 23.9% nonusers).

### Study Instruments

The questionnaire used in this study was adapted from a previously validated instrument based on a modified TAME-ChatGPT (Technology Acceptance Model Edited to Assess ChatGPT Adoption), which demonstrated acceptable reliability in prior studies (Cronbach α≥0.71) [21]. In this study, the adapted questionnaire was pilot-tested to assess clarity and comprehension before deployment, as shown in Multimedia Appendix 1.

The survey measured ChatGPT perception on four constructs that corresponded to the research aim: (1) perceived usefulness, which reflects participants’ beliefs that ChatGPT is useful for accessing and supporting health-related information; (2) perceived risk, which includes threats related to privacy, security, and reliability when accessing and supporting health-related information; (3) anxiety, which refers to fear or worry about using ChatGPT for health-related purposes; and (4) social influence, which captures the perceived influence of peers or family on participants’ readiness or recommendation to use the tool.

All items were evaluated using a 5-point Likert scale (from “strongly disagree” to “strongly agree”), with higher summed scores of relevant items indicating greater support for the construct.

Sociodemographic information and health-related characteristics were also collected in the questionnaire, considering the status of ChatGPT users and nonusers who were aware of ChatGPT but had not used it. This method enabled comparison of perceptions and attitudes toward ChatGPT between user and nonuser groups in the context of health-related decision-making.

### Study Techniques, Duration, and Setting

Data collection was conducted from mid-November 2023 to February 2024 using a convenience sampling technique. Participants were recruited via official university email systems and social media platforms. Email invitations were distributed across Imam Abdulrahman bin Faisal University (Eastern province), Umm Al-Qura University (Makkah province), Shaqra University, and Prince Sultan University (Riyadh province) to capture diverse geographic locations and perspectives. Additional invitations were shared via Telegram (12.6.2) and WhatsApp (version ; Meta Platforms Inc) groups.

### Statistical Analysis

In this study, SPSS (version 23.0; IBM Corp) was used for all data analysis. Descriptive statistics, including frequencies, percentages, means, and SD, were used to summarize the characteristics of participants and the overall scores of perception. Additionally, the *χ*^2^ test was used to evaluate relationships between ChatGPT use status (users vs nonusers) and sociodemographic variables. Furthermore, factor scores were summarized using descriptive statistics, including means (SDs), medians (IQRs), and ranges, to describe the central tendency and dispersion of responses, as the primary goal was to characterize the distribution of perceptions across factors. Both mean (SD) and median (IQR) were reported for all perception factor scores to consistently summarize central tendency and dispersion and to provide a complete description of their distribution, regardless of normal distribution. The level of statistical significance was set at *P*≤.05.

### Ethical Considerations

The institutional review board of Imam Abdulrahman bin Faisal University granted ethics approval (IRB-HAP-05-D-003) for this study. All participants provided informal consent before completing the survey. Participant privacy and confidentiality were maintained, with all data anonymized and access restricted to the research team. No financial compensation was provided to participants.

## Results

Among the analytic sample of 255 participants, 194 (76.1%) were ChatGPT users and 61 (23.9%) were nonusers who were familiar with ChatGPT. Participant sociodemographic characteristics are presented in Table 1. Overall, 89.8% (n=229) were Saudi nationals, and 53.7% (n=137) had a bachelor’s degree. The participants’ family income varied; approximately half (n=125, 49%) reported earning ≥15,000 SAR 3.75 (US $1.00). Among the participants, 63.5% (n=162) were single, 35.3% (n=90) were married, and 1.2% (n=3) were divorced or widowed. In terms of regions, the majority of participants (n=119, 46.7%) were in the central region, followed by the western (n=65, 25.5%) and eastern regions (n=48, 18.8%), with no substantial difference between the southern (n=11, 4.3%) and the northern (n=12, 4.7%) regions.

**Table 1.** Sociodemographic characteristics of the participants (N=255).

Sociodemographic characteristics	Participants, n (%)
Sex	
Female	166 (65.1)
Male	89 (34.9)
Age group (years)	
18-24	112 (43.9)
25-34	67 (26.3)
35-44	55 (21.6)
≥45	21 (8.2)
Nationality	
Saudi	229 (89.8)
Non-Saudi	26 (10.2)
Educational level	
Elementary and secondary	28 (11.0)
Bachelor’s degree	137 (53.7)
Master’s degree	55 (21.6)
Doctorate	35 (13.7)
Family income (SAR; US $1.00=SAR 3.75)	
<1000	13 (5.1)
1000-2999	11 (4.3)
3000-5999	17 (6.7)
6000-8999	27 (10.6)
9000-11,999	34 (13.3)
12,000-14,999	28 (11.0)
≥15,000	125 (49.0)
Marital status	
Single	162 (63.5)
Married	90 (35.3)
Divorced or widowed	3 (1.2)
Region	
Central	119 (46.7)
Western	65 (25.5)
Eastern	48 (18.8)
Southern	11 (4.3)
Northern	12 (4.7)
Have you used ChatGPT?	
Yes	194 (76.1)
No	61 (23.9)

**Figure 1.**
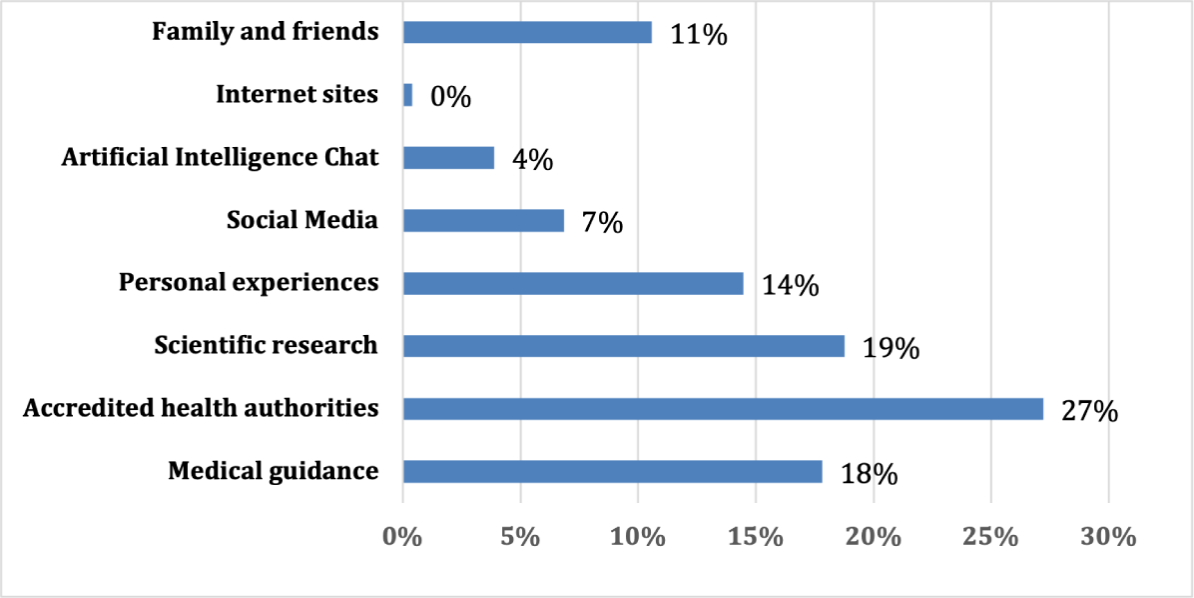
Source of clinical information.

In total, 194 (76%) participants reported using ChatGPT. When participants were asked about their sources of health information, the majority (n=69, 27%) reported health authorities, whereas only 4% (n=10) reported AI-based sources. Furthermore, personal experience, scientific sources, and medical guidance were reported by 10% (n=36) to 20% (n=48) of participants as sources of health information (Figure 1).

**Figure 2.**
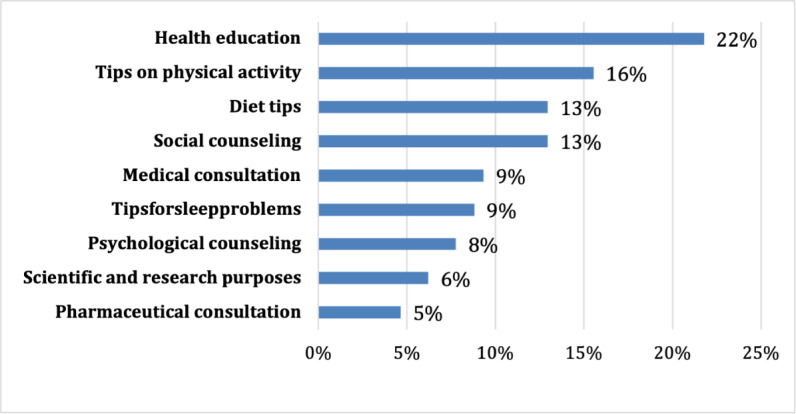
Percentage of ChatGPT users by main health-related purpose.

Figure 2 shows that 22.2% (43/194) of ChatGPT users reported using it for health education, followed by 16% (n=31) using it for tips on physical activity. However, use for scientific and research purposes, psychological counseling, tips for sleep problems, and medical consultation ranged from 5% (n=10) to 10% (n=17), while use for diet tips and social counseling ranged from 10% (n=25) to 15% (n=31).

Female participants represented a higher percentage of ChatGPT users than male participants (127/194, 65.5% vs 67/194, 34.5%, respectively), and participants aged <24 years had the highest use rate (87/194, 44.8%) compared to other age groups (Table 2). Respondents with bachelor’s degrees (98/194, 50.5%) reported using ChatGPT more than other degree holders. Moreover, those who were single reported higher use of ChatGPT than those who were married, divorced, or widowed (124/194, 63.9%; 68/194, 35.1%; and 2/194, 1%, respectively). In contrast, unemployed participants (105/194, 54.1%) reported higher use of ChatGPT than employed participants (89/194, 45.9%). However, there were no significant associations between socioeconomic variables and ChatGPT use.

**Table 2.** Comparison of sociodemographic characteristics between ChatGPT users and nonusers (N=255).

Characteristics	User, n (%)	Nonuser, n (%)	*χ*^2^ (*df*)	*P* value
Sex			0.048 (1)	.83
Female	127 (65.5)	39 (63.9)		
Male	67 (34.5)	22 (36.1)		
Age group (years)			0.487 (3)	.92
18-24	87 (44.85)	25 (40.98)		
25-34	50 (25.77)	17 (27.87)		
35-44	42 (21.65)	13 (21.31)		
≥45	15 (7.73)	6 (9.84)		
Educational level			4.355 (3)	.23
Elementary and secondary	21 (10.82)	7 (11.48)		
Bachelor’s degree	98 (50.52)	39 (63.93)		
Master’s degree	45 (23.20)	10 (16.39)		
Doctorate	30 (15.46)	5 (8.20)		
Marital status			0.179 (2)	.91^a^
Single	124 (63.92)	38 (62.30)		
Married	68 (35.05)	22 (36.06)		
Divorced or widowed	2 (1.03)	1 (1.64)		
Employment status			0.455 (1)	.50
Not employed	105 (54.12)	30 (49.18)		
Employed	89 (45.88)	31 (50.82)		
Family income (Saudi Riyal)			4.309 (6)	.64^a^
<1000	11 (5.67)	2 (3.28)		
1000-2999	8 (4.12)	3 (4.92)		
3000-5999	15 (7.73)	2 (3.28)		
6000-8999	21 (10.83)	6 (9.84)		
9000-11,999	25 (12.89)	9 (14.75)		
12,000-14,999	18 (9.28)	10 (16.39)		
≥15,000	96 (49.48)	29 (47.54)		

a Significance level set at *P*≤.05.

Table 3 shows users’ perspectives on using ChatGPT. In total, 83.5% (162/194) of participants reported favorable responses, 11.9% (23/194) reported neutral responses, and 4.6% (9/194) reported unfavorable responses, indicating that ChatGPT is perceived as quick and easy to learn. Furthermore, the results indicate 45.9% (89/194) favorable responses, 30.9% (60/194) neutral responses, and 23.2% (45/194) unfavorable responses, illustrating that a higher proportion believed that ChatGPT is helpful for saving time when searching for health information. In contrast, when recommending ChatGPT to family and colleagues for accessing health information, 26.3% (51/194) of participants reported favorable responses, 27.8% (54/194) reported neutral responses, and 45.9% (89/194) reported unfavorable responses. Moreover, only 20.6% (40/194) favorable responses (50/194, 25.8% neutral and 104/194, 53.6% unfavorable responses) reflect that ChatGPT was perceived as more beneficial than other health information sources. A notable proportion of participants reflected on the accuracy and reliability of the health information provided by ChatGPT, with 25.2% (49/194) reporting favorable responses, 29.9% (58/194) reporting neutral responses, and 44.9% (87/194) reporting unfavorable responses. Nonetheless, participants agreed that security risks (109/194, 56.2% favorable; 33/194, 17% neutral; and 2/194, 26.8% unfavorable responses) and privacy risks were concerns when using ChatGPT (106/194, 54.7% favorable; 44/194, 22.7% neutral; and 44/194, 22.7% unfavorable responses). Furthermore, a certain proportion believed that it could lead to misinformation (68/194, 35.1% favorable; 85/194, 43.8% neutral; and 41/194, 21.2% unfavorable responses), and most users did not automatically use it for medical decisions (32/194, 16.5% favorable; 39/194, 20.1% neutral; and 123/194, 63.4% unfavorable responses). However, most believed that ChatGPT cannot reduce their visits to a physician (39/194, 20.1% favorable; 29/194, 14.9% neutral; and 126/194, 65% unfavorable responses).

**Table 3.** ChatGPT users’ perspective on health-related attitudes^a^.

Statements	Strongly disagree, n (%)	Disagree, n (%)	Neutral, n (%)	Agree, n (%)	Strongly agree, n (%)
ChatGPT helps me save time when searching for health information.	19 (9.8)	26 (13.4)	60 (30.9)	51 (26.3)	38 (19.6)
I recommend ChatGPT to my family and colleagues to access health information.	32 (16.5)	57 (29.4)	54 (27.8)	32 (16.5)	19 (9.8)
ChatGPT is more useful than other health information sources I have previously used.	44 (22.7)	60 (30.9)	50 (25.8)	27 (13.9)	13 (6.7)
I appreciate the accuracy and reliability of health information provided by ChatGPT.	36 (18.6)	51 (26.3)	58 (29.9)	42 (21.6)	7 (3.6)
I believe that using ChatGPT can help save the time and effort required to make health decisions.	30 (15.5)	36 (18.6)	48 (24.7)	57 (29.4)	23 (11.9)
I have used tools or techniques similar to ChatGPT in the past.	20 (10.3)	54 (27.8)	29 (14.9)	62 (32.0)	29 (14.9)
I automatically find myself using ChatGPT when I need information to make a health decision.	52 (26.8)	71 (36.6)	39 (20.1)	23 (11.9)	9 (4.6)
I often use ChatGPT as a source of health information.	64 (33.0)	71 (36.6)	27 (13.9)	23 (11.9)	9 (4.6)
Using ChatGPT leads to misleading information.	4 (2.1)	37 (19.1)	85 (43.8)	56 (28.9)	12 (6.2)
I am concerned about the potential security risks of using ChatGPT.	11 (5.7)	41 (21.1)	33 (17.0)	64 (33.0)	45 (23.2)
I believe that relying on technology such as ChatGPT can reduce my visits to a physician.	56 (28.9)	70 (36.1)	29 (14.9)	28 (14.4)	11 (5.7)
It takes a short time to learn how to use ChatGPT.	3 (1.5)	6 (3.1)	23 (11.9)	73 (37.6)	89 (45.9)
There are potential privacy risks associated with using ChatGPT.	8 (4.1)	36 (18.6)	44 (22.7)	70 (36.1)	36 (18.6)

a Unfavorable (strongly disagree+disagree), neutral, and favorable (agree+strongly agree) categories are presented for descriptive purposes. Percentages indicate trends and do not imply statistical significance.

Table 4 presents the perspectives of nonusers on ChatGPT. Concerns included lack of confidence in information reliability (11/61, 18.1% favorable; 23/61, 37.7% neutral; and 27/61, 44.3% unfavorable responses), security risks (39/61, 64% favorable; 14/61, 23% neutral; and 8/61, 13.1% unfavorable responses), privacy risks (36/61, 59% favorable; 19/61, 31.1% neutral; and 6/61, 9.8% unfavorable responses), and misinformation (24/61, 39.4% favorable; 29/61, 47.5% neutral; and 8/61, 13.1% unfavorable responses). Similar to the users, nonusers believed that ChatGPT could not reduce physician visits (23/61, 37.7% favorable; 6/61, 9.8% neutral; and 32/61, 52.4% unfavorable responses) and should not be relied on for making health decisions (11/61, 18% favorable; 8/61, 13.1% neutral; and 42/61, 68.8% unfavorable responses). However, nonusers expressed interest in using ChatGPT to search for health information (30/61, 49.2% favorable; 13/61, 21.3% neutral; and 18/61, 29.5% unfavorable responses) and recognized it as an important tool for accessing such information (23/61, 37.7% favorable; 20/61, 32.8% neutral; and 18/61, 29.5% unfavorable responses). Additionally, they believed that technologies such as ChatGPT are attractive and fun (42/61, 68.9% favorable; 14/61, 23% neutral; and 5/61, 8.2% unfavorable responses) and were willing to learn how to use it (36/61, 59% favorable; 19/61, 31.1% neutral; and 6/61, 9.8% unfavorable responses). Trust in the opinions of family and colleagues plays a role in participants’ willingness to adopt ChatGPT, with 44.2% (27/194) reporting favorable responses, 39.3% (24/194) reporting neutral responses, and 16.4% (10/194) reporting unfavorable responses.

**Table 4.** Non-ChatGPT users’ perspectives on ChatGPT for health-related attitudes^a^.

Statements	Strongly disagree, n (%)	Disagree, n (%)	Neutral, n (%)	Agree, n (%)	Strongly agree, n (%)
I am confident about the reliability of the health information provided by ChatGPT.	13 (21.3)	14 (23)	23 (37.7)	9 (14.8)	2 (3.3)
Using ChatGPT leads to misleading information.	0 (0)	8 (13.1)	29 (47.5)	17 (27.9)	7 (11.5)
There are potential security risks associated with using ChatGPT.	0 (0)	8 (13.1)	14 (23)	30 (49.2)	9 (14.8)
There are potential privacy risks associated with using ChatGPT.	0 (0)	6 (9.8)	19 (31.1)	19 (31.1)	17 (27.9)
Relying on technology such as ChatGPT can reduce my visits to a physician.	13 (21.3)	19 (31.1)	6 (9.8)	19 (31.1)	4 (6.6)
I may rely entirely on technology such as ChatGPT to make my health decisions.	19 (31.1)	23 (37.7)	8 (13.1)	8 (13.1)	3 (4.9)
I am passionate about using technology such as ChatGPT to learn and search for health information.	3 (4.9)	15 (24.6)	13 (21.3)	27 (44.3)	3 (4.9)
I believe that technology such as ChatGPT is an important tool for accessing health information.	7 (11.5)	11 (18)	20 (32.8)	20 (32.8)	3 (4.9)
I think that technology such as ChatGPT is attractive and fun to use.	0 (0)	5 (8.2)	14 (23.0)	35 (57.4)	7 (11.5)
I am always keen to learn about new technologies such as ChatGPT.	0 (0)	6 (9.8)	19 (31.1)	29 (47.5)	7 11.5
I trust the opinions of my family or colleagues about using ChatGPT.	1 (1.6)	9 (14.8)	24 (39.3)	21 (34.4)	6 (9.8)

a Unfavorable (strongly disagree+disagree), neutral, and favorable (agree+strongly agree) categories are presented for descriptive purposes. Percentages indicate trends and do not imply statistical significance.

Table 5 presents participants’ perceptions regarding ChatGPT use across the main constructs. ChatGPT users (n=194) reported their scores on perceived usefulness, behavioral cognitive aspects, perceived risk of use, and perceived ease of use. The levels of perceived risk, anxiety, and technology or social influence were reported in nonusers who were knowledgeable about ChatGPT (n=61). Overall, participants reported moderate perceived usefulness (mean 7.2, SD 3.9), indicating general agreement that the technology is beneficial. However, ease of use was rated lower (mean 5.3, SD 1.5), suggesting that while some found the system accessible, others faced usability challenges. Perceived risk was notably high (mean 8.4, SD 3.0), and anxiety associated with ChatGPT use was also present (mean 2.9, SD 2.1), indicating potential hesitancy or concerns regarding the technology. Interestingly, responses from participants who reported unfamiliarity with ChatGPT (n=61) showed an even higher perceived risk (mean 9.4, SD 2.1), reinforcing the idea that unfamiliarity contributes to skepticism. The role of technology and social influence emerged as a positive factor (mean 11.9, SD 3.2), suggesting that external influences, such as peer recommendations or societal trends, play a significant role in shaping perceptions toward ChatGPT. Similarly, behavioral cognitive factors showed moderate endorsement (mean 4.6, SD 2.6), indicating variability in participants’ attitudes toward adoption.

**Table 5.** Participants’ perceptions regarding the use of ChatGPT across various factors^a^.

Measures	Users				Nonusers		
	Perceived usefulness	Behavioral cognitive factor	Perceived risk of use	Perceived ease of use	Perceived risk	Anxiety	Technology or social influence
Mean (SD)	7.2 (3.9)	4.6 (2.6)	8.4 (3.0)	5.3 (1.5)	9.4 (2.1)	2.9 (2.1)	11.9 (3.2)
Median (IQR)	7.5 (4-10)	5.0 (3-6)	8.0 (6-10)	5.0 (4-6)	9.0 (8-11)	3.0 (1-4)	12.0 (10-14)
Range	0-16	0-12	2-16	0-8	3-14	0-8	2-20

a Factor scores were calculated by summing participants’ responses to all items within each factor. Items were rated on a 5-point Likert scale (0=strongly disagree to 4=strongly agree). Total scores for each factor were then used to compute descriptive statistics, including mean (SD), median (IQR), and range. Sample sizes were n=194 for users and n=61 for nonusers of ChatGPT.

## Discussion

### Principal Findings

This study analyzed views on ChatGPT as a health information–seeking tool among users and nonusers in Saudi Arabia. The findings indicate that most participants reported traditional health authorities as their most frequently cited sources of health information. The use of ChatGPT differed across participants, with some reporting its use for health education and others for physical activity. Fewer participants reported using it for psychological counseling, sleep-related problems, and medical consultation. Although this has recently become a topic of discussion, research indicates progress in this area [22]. However, nutrition advice and social counseling were found to be more effective. This aligns with studies suggesting that AI-powered chatbots can significantly improve dietary guidance [23]. These results indicate that ChatGPT might not yet be a substitute for traditional health information sources but rather an additional source of health-related information.

It has been noted that the use of ChatGPT was common across different sociodemographic groups, and differences between users and nonusers were not found to be significant. Younger participants (age ≤24 years) and those with doctoral degrees were slightly more likely to be using ChatGPT; however, these differences were not statistically significant. According to previous research, higher educational and socioeconomic levels may also affect the use of AI-based applications such as ChatGPT [24-26]. Although age has been reported to influence willingness to embrace AI technologies [27], this effect was not observed in this study. ChatGPT was used for health education and instructions on physical activity; however, the study did not evaluate whether the information prompted actual behavior change.

Prior research investigating perceptions of usefulness, ease of use, risk, and psychosocial issues has indicated a generally positive attitude toward ChatGPT [28]. Other studies involving health care professionals and students have yielded similar results, with perceived usefulness moderated by reliability and perceived risks [12,29]. These results are consistent with this study, in which respondents reported moderate perceived usefulness of ChatGPT and concerns about privacy, security, and misinformation. Although prior studies indicated that social and age-related variables could contribute to the adoption of AI technologies [30,31], the data in this study did not show statistically significant relationships between age and ChatGPT use.

In this study, a large number of ChatGPT users found it useful and relatively easy to use, but very few said that they used it when making health-related decisions. There were also concerns regarding the accuracy, privacy, and security of information, especially among nonusers. These issues align with previous studies showing that users remain skeptical about trusting AI-generated information and about using it in medical practice [32,33].

Even though numerous participants were interested in technologies such as ChatGPT and reported positive expectations of its usefulness, the findings should be interpreted in light of the descriptive nature of the data. Perceived usefulness was more likely to be accentuated among users, whereas perceived risk was more relevant among nonusers.

This study has a number of limitations. First, the cross-sectional design does not permit any causal inference. Second, the use of convenience sampling can introduce selection bias and reduce the representativeness of the sample. Third, the study used self-reported data, which could be affected by recall bias, reporting bias, and social desirability bias.

Despite these limitations, this study provides insight into people’s perception of ChatGPT as a health information–seeking tool. Although the participants were aware of its potential usefulness, concerns regarding privacy, security, and information reliability remained important considerations. Future studies should consider using more diverse and representative samples and investigating how perceptions of AI-based health information tools change over time.

### Conclusions

The study investigated perceptions of ChatGPT as a health information–seeking tool among users and nonusers in Saudi Arabia. ChatGPT was viewed as a convenient and accessible source of health-related information, especially for health education and assistance with physical activity. However, concerns regarding privacy, security, and reliability of information were raised by both users and nonusers. These findings indicate that interest in AI-based tools such as ChatGPT exists, but issues related to trust, data privacy, and information quality can arise when they are used to seek health-related information.

There is a need for further investigation into AI-driven health care decision-making, particularly regarding privacy, trust, and long-term adoption patterns. Policymakers should focus on enhancing security measures, ensuring accuracy, and fostering trust to facilitate the responsible use of AI tools in the health care system.

## Supplementary material

10.2196/79276 Multimedia Appendix 1 Questionnaire items.

## Abbreviations

AI: artificial intelligence
TAME-ChatGPT: Technology Acceptance Model Edited to Assess ChatGPT Adoption
