# Supplementary material for: Comparing Perceptions of ChatGPT Use in Health Attitude Contexts Among Users and Nonusers: Cross-Sectional Study
Source: JMIR Form Res. 2026 Apr 27;10:e79276. doi: 10.2196/79276 (PMC13119390; doi:10.2196/79276)
Supplement: Multimedia Appendix 1 [file formative-v10-e79276-s001.docx]

ChatGPT Questions: (5 points Scores per question)

**Usage Scale**

**A. Perceived Usefulness**

1. "**ChatGPT helps me save time when searching for health information**."
   → Highlights the time-saving benefit, reflecting usefulness.
2. "**I recommend ChatGPT to my family and colleagues to access health information**."
   → Suggests perceived value and effectiveness, indicating usefulness.
3. "**ChatGPT is more useful than other health information sources you have previously used**."
   → Directly measures comparative utility.
4. "**I appreciate the accuracy and reliability of the health information provided by ChatGPT**."
   → Relates to trust and perceived quality, key aspects of usefulness.

**B. Behavior/Cognitive Factors**

1. "**I have used tools or techniques similar to ChatGPT in the past**."
   → Reflects prior behavior influencing adoption.
2. "**I automatically find myself using ChatGPT when I need information to make a health decision**."
   → Measures habitual behavior and cognitive reliance.
3. "**I often use ChatGPT as a source of health information**."
   → Captures frequency of use, a core behavioral indicator.

**C. Perceived Risk of Use**

1. "**Using ChatGPT leads to misleading information**."
   → Reflects concerns about accuracy, a key risk factor.
2. "**I am concerned about the potential security risks of using ChatGPT**."
   → Highlights user anxiety regarding security.
3. "**There are potential privacy risks of using ChatGPT**."
   → Addresses concerns about data privacy and trust.
4. "**I believe that relying on technology such as ChatGPT can reduce my visits to the doctor**."
   → Indicates perceived risk of over-reliance on technology for critical decisions.

**D. Perceived Ease of Use**

1. "**It takes a short time to learn to use ChatGPT**."
   → Relates directly to ease of learning and adoption.
2. "**I believe that using ChatGPT can help save the time and effort required to make health decisions**."
   → Emphasizes that using ChatGPT reduces the complexity, integral to perceived usefulness and indicators of ease of use.

**Heard of ChatGPT but Did Not Use It**

**A. Perceived Risk**

1. "**I am confident about the reliability of the information provided by ChatGPT**."
   → Opposite of perceived risk; addresses **reliability** but indirectly reflects concerns about accuracy.
2. "**Using ChatGPT leads to misleading information**."
   → Fits directly under **perceived risk** as it questions the accuracy and potential harm of incorrect information.
3. "**There are potential security risks to using ChatGPT**."
   → Aligns with **concerns about security** risks, as explicitly stated in the framework.
4. "**There are potential privacy risks of using ChatGPT**."
   → Directly reflects **concerns about privacy** risks.

**B. Anxiety**

1. "**I may rely entirely on technology such as ChatGPT to make my health decisions**."
   → Reflects anxiety about **over-reliance** on ChatGPT, leading to diminished personal or critical thinking skills.
2. "**Relying on technology such as ChatGPT can reduce my visits to the doctor**."
   → Demonstrates potential anxiety over substitution for professional judgment with AI reliance.

**C. Technology/Social Influence**

1. "**I am passionate about using technology like ChatGPT to learn and search for health information**."
   → Reflects **enthusiasm** about learning and using innovative technologies.
2. "**I believe that technology such as ChatGPT is an important tool for accessing health information**."
   → Highlights **belief in technology's importance** for academic or research success.
3. "**I think technology like ChatGPT is attractive and fun to use**."
   → Captures **perceived attractiveness** and usability of ChatGPT.
4. "**I'm always keen to learn about new technologies like ChatGPT**."
   → Demonstrates **curiosity and enthusiasm** for exploring new tech.
5. "**I trust the opinions of my family or colleagues about using ChatGPT**."
   → Reflects **social influence** and trust in others' opinions about using ChatGPT.

**General Questions:**

**Demographics**

- Sex
- Age
- Nationality
- Educational level
- Marital status
- Professional situation (Which of the following describes your professional situation?)
- Total family income in riyals (includes the salaries of all individuals in the house)
- Place of residence
- Region
- Are you a health practitioner (doctor, nurse, pharmacist, etc.)?

**Health Related Characteristic**

1. **Health Status**

- Smoking status
- Physical health status (Which of the following describes your physical health?)
- Mental health status (How would you describe your mental health in general?)
- Overall anxiety and stress levels (5 being the best and 0 being the worst)
- Are you a person with special needs?
- Do you have health insurance?
- Do you use any medications or nutritional supplements?
- How accessible is healthcare?

1. **Lifestyle Habits**

- How many days do you engage in sports or intense physical activity for 30 minutes or more?
- Best description of your diet
- Sleep duration

**Technology Usage**

- What is ChatGPT known as?
- Have you ever used ChatGPT?
- How often do you use ChatGPT?
- What device is mostly used for ChatGPT?

**Health Information Preferences**

- Where do you rely on to make health information and decisions? (multiple choices)
- Have you used ChatGPT for health purposes such as diagnosis, diet, medical, or pharmaceutical advice, etc.?
